# Abnormal Meiosis Initiation in Germ Cell Caused by Aberrant Differentiation of Gonad Somatic Cell

**DOI:** 10.1155/2019/8030697

**Published:** 2019-09-05

**Authors:** Min Chen, Min Chen, Suren Chen, Jingjing Zhou, Fangfang Dong, Zhiming Shen, Haowei Wu, Xiuhong Cui, Fei Gao

**Affiliations:** ^1^State Key Laboratory of Stem cell and Reproductive Biology, Institute of Zoology, Chinese Academy of Sciences, Beijing 100101, China; ^2^University of Chinese Academy of Sciences, Beijing 100049, China

## Abstract

The interaction between germ cell and somatic cell plays important roles in germ cell development. However, the exact function of gonad somatic cell in germ cell differentiation is unclear. In the present study, the function of gonad somatic cell in germ cell meiosis was examined by using mouse models with aberrant somatic cell differentiation. In *Wt1^R394W/R394W^* mice, the genital ridge is absent due to the apoptosis of coelomic epithelial cells. Interestingly, in both male and female *Wt1^R394W/R394W^* germ cells, STRA8 was detected at E12.5 and the scattered SYCP3 foci were observed at E13.5 which was consistent with control females. In *Wt1^-/flox^; Cre-ER^TM^* mice, *Wt1* was inactivated by the injection of tamoxifen at E9.5 and the differentiation of Sertoli and granulosa cells was completely blocked. We found that most germ cells were located outside of genital ridge after *Wt1* inactivation. STRA8, SYCP3, and *γ*H2AX proteins were detected in germ cells of both male and female *Wt1^-/flox^; Cre-ER^TM^* gonads, whereas no thread-like SYCP3 signal was observed. Our study demonstrates that aberrant development of gonad somatic cells leads to ectopic expression of meiosis-associated genes in germ cells, but meiosis was arrested before prophase I. These results suggest that the proper differentiation of gonad somatic cells is essential for germ cell meiosis.

## 1. Introduction

In mice, primordial germ cells (PGCs) arise from extraembryonic ectoderm at approximately E6.25 and migrate to the developing genital ridge at E10.5 [1]. After several rounds of mitosis, the germ cells in male gonads enter G0/G1 arrest between E12.5 and E14.5 until initiating meiosis after birth. The female germ cells start meiosis right after sex determination at approximately E12.5, then arrest at diplotene stage of prophase I until ovulation and the meiosis is completed after fertilization [2]. The different fates of germ cells in male and female gonads are not determined by the sex chromosome constitution but by the somatic cells in the gonad [3].

Retinoic acid (RA) is the most important extrinsic factor which is indispensable for germ cell meiosis initiation. RA is synthesized in mesonephros and diffuses into adjacent gonad to induce the expression of *Stra8* in germ cells of female gonads [4–7]. In male gonads, the germ cells are surrounded by Sertoli cells in testicular cords. Cytochrome P450, family 26, subfamily b, polypeptide 1 (*Cyp26b1*), is highly expressed in Sertoli cells during embryonic stages, which catalyzes the oxidization of RA to inactive metabolites. Therefore, the germ cells in male gonad could not access RA and initiate meiosis during embryonic stage.

As a nuclear transcription factor, Wilms tumor gene 1 (*Wt1*) is abundantly expressed in the coelomic epithelium of the urogenital ridge and the underlying mesenchymal cells before sex determination [8]. In sex-committed gonads, *Wt1* is specifically expressed in both Sertoli cells and granulosa cells. *WT1* is originally identified as a tumor suppressor gene associated with the development of Wilms' tumors and is subsequently found to be mutated in patients with Denys-Drash syndrome (DDS) [9]. *Wt1^R394W/R394W^* mice are embryonic lethal and the genital ridge can not develop [10]. Our previous study demonstrates that in *Wt1^R394W/R394W^* mice, the directional migration of PGCs is not affected and most PGCs reach the mesenchyme under the coelomic epithelium at E10.5 which is consistent with control embryos [11]. We also find that when *Wt1* is deleted at approximately E9.5 using *Cre-ER^TM^*, the development of genital ridge is not affected, whereas the differentiation of both Sertoli and granulosa cells is blocked and most genital ridge somatic cells differentiate into steroidogenic cells in both male and female gonads [12].

It has been proposed that the differentiation of gonad somatic cell plays important roles in germ cell development. However, the exact functions of somatic cells are still unclear. In this study, the function of somatic cell on germ cell meiosis is examined by using genital agenesis (*Wt1^R394W/R394W^*) and somatic aberrantly differentiated (*Wt1^-/flox^; Cre-ER^TM^*) mouse models. We find both male and female germ cells start to express STRA8, but no germ cells at prophase I are observed. Our study demonstrates that the meiosis initiation of germ cell is accurately regulated by somatic factors.

## 2. Materials and Methods

### 2.1. Mice

All animal work was carried out in accordance with institutional animal care and the use committee regulations of Institute of Zoology, CAS. All mice were maintained in a C57BL/6;129/SvEv mixed background. The mouse strain carrying the *Wt1^R394W^* point mutation was generated in Dr. Vicki Huff's laboratory [10]. *Wt1^R394W/R394W^* mice were obtained by crossing male and female *Wt1^+/R394W^* mice. *Wt1^-/flox^; Cre-ER^TM^* offspring were obtained by crossing *Wt1^flox/flox^* mice with *Wt1^+/−^* and *Cre-ER^TM^* transgenic mice. DNA isolated from adult tails and fetal tissues was used for genotyping. Pregnant *Wt1^flox/flox^* females were injected with Tamoxifen (Sigma-Aldrich) intraperitoneally at a dose of 6 mg/40 g body weight at E9.5 to induce Cre activity as described previously [12]. *Wt1^flox/flox^* and *Wt1^-/flox^* embryos were used as controls.

### 2.2. Organ Culture

The experiment of organ culture was performed as described previously [13, 14]. In brief, pregnant *Wt1^flox/flox^* females were injected with tamoxifen at E9.5. The gonads with mesonephroi were dissected from control and *Wt1^-/flox^; Cre-ER^TM^* embryos at E13.5, placed on agarose stands (1.5% *w*/*v*, in 24-well plates), and cultured at 37°C and 5% CO_2_. After 3 days of culture, the gonads were fixed in 4% PFA for further analysis.

### 2.3. Tissue Collection and Histological Analysis

Control and *Wt1*-inactivated embryos were collected immediately following euthanasia of pregnant mice. Gonads with mesonephroi were dissected in PBS, fixed in 4% paraformaldehyde for up to 24 hrs, stored in 70% ethanol, and embedded in paraffin. Then, tissue sections of 5 *μ*m thickness were cut and mounted on glass slides.

### 2.4. Immunofluorescence Analysis and TUNEL Assay

Tissue sections were deparaffinized, rehydrated, and subjected to antigen retrieval. After blocking in 5% donkey serum in 0.3% Triton X-100 for 1 hr, the sections were incubated with primary antibodies for 1.5 hrs and the corresponding FITC-conjugated and Cy^TM^3-conjugated secondary antibodies (1 : 150 and 1 : 300, respectively, Jackson) for 1 hr at room temperature. The following dilutions of primary antibodies were used: STELLA (1 : 200, Santa Cruz, sc-67249), GATA4 (1 : 300, Santa Cruz, sc-1237), STRA8 (1 : 200, Abcam, ab49405), SYCP3 (1 : 200, Abcam, ab15093), *γ*H2AX (1 : 200, Millipore, 05-636), DAZL (1 : 100, AbD Serotec, MCA2336), and MVH (1 : 500, Abcam, ab13840). After three washes in PBS, the sections were counterstained with DAPI to label the nuclei. The images were captured with a confocal laser scanning microscope (Carl Zeiss Inc., Thornwood, NY). TUNEL assay was conducted using the DeadEnd Fluorometric TUNEL system (Promega, G3250) as recommended.

### 2.5. Quantitative Reverse Transcription PCR

Gonads of E13.5 embryos were used to extract total RNA using a Qiagen RNeasy kit following manufacturer's instructions. The relative expression level was calculated using the formula 2^−ΔΔCT^. *Hprt1* was used as an endogenous control. The primers used were listed as follows: *Stra8* sense, CTCCTCCTCCACTCTGTTGC, antisense, GCGGCAGAGACAATAGGAAG; *Sycp3* sense, AGAAATGTATACCAAAGCTTCTTTCAA, antisense, TTAGATAGTTTTTCTCCTTGTTCCTCA; *Rec8* sense, CTACCTAGCTTGCTTCTTCCCA, antisense, GCCTCTAAAAGGTGTCGAATCTG; and *Dmc1* sense, CCCTCTGTGTGACAGCTCAAC, antisense, GGTCAGCAATGTCCCGAAG.

### 2.6. Chromosome Spread and Immunofluorescence

After culture for three days, the gonads were incubated in hypotonic extraction buffer (30 mM Tris, pH 8.2; 50 mM sucrose; 17 mM trisodium citrate dihydrate; 5 mM EDTA; 0.5 mM DTT; and 0.5 mM PMSF) for 45 mins at room temperature. After hypotonic treatment, 100 *μ*l sucrose (100 mM) was added and cell suspension was pipetted up and down for several times. APES treated slides were coated with 1% paraformaldehyde containing 0.15% Triton X. 10 *μ*l cell suspension was dispersed to the slide containing a layer of paraformaldehyde. Slides were placed in a humid chamber for at least 6 hrs at room temperature, then allowed to air dry and stored at −80°C until use.

The slides were washed in 0.4% Kodak Photo-Flo 200 for 4 min and 0.1% Triton X-100 in PBS for three times, blocked in 200 *μ*l blocking buffer (3% nonfat milk in PBST) for 1 hr at room temperature, followed by an overnight incubation with primary antibody at 4°C and the corresponding FITC-conjugated and Cy^TM^3-conjugated secondary antibodies for 1 hr. After three washes in PBS, the sections were analyzed with a confocal laser scanning microscope (Carl Zeiss Inc., Thornwood, NY).

### 2.7. Statistical Analysis

Experiments were repeated at least three times. Three to five control, *Wt1*-mutant or *Wt1*-deficient male or female embryos at each time point were used for immunostaining. For gonad culture, at least 4 pairs of male or female gonads of each genotype were used. For real-time PCR, 3 pairs of gonads of the same genotype were pooled and three independent pools were used for RNA preparation. The results are presented as the mean ± SEM. Student's *t*-test was used to analyze the data. Probability values of <0.05 were considered as significant.

## 3. Results and Discussion

### 3.1. *STRA8* Was Expressed in Both Male and Female Germ Cells of *Wt1^R394W/R394W^* Mice

**Figure 1:**
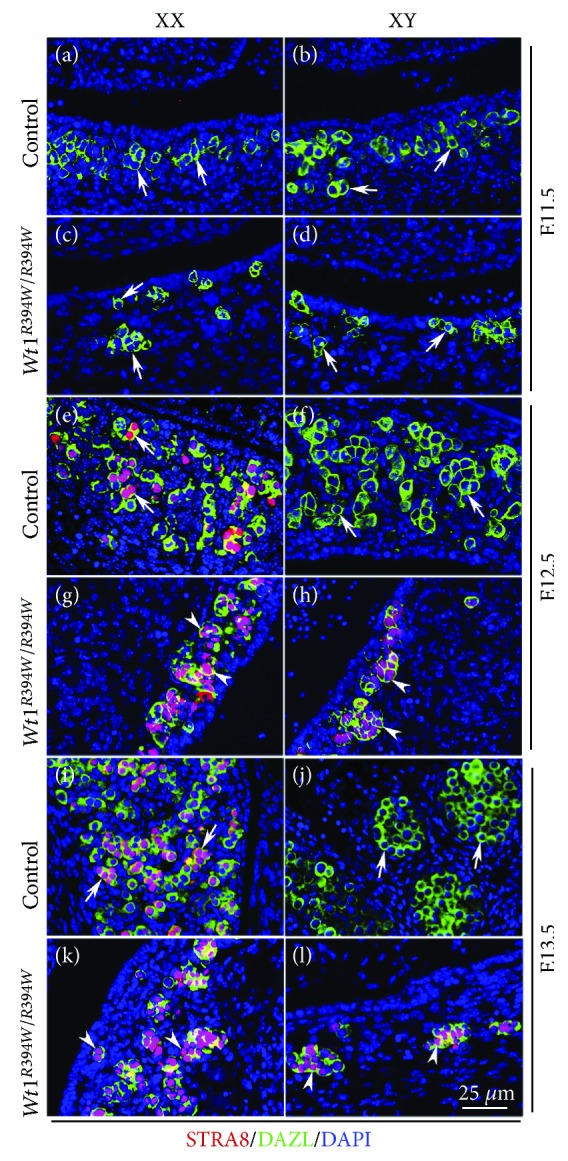
STRA8 was expressed in germ cells of both male and female *Wt1^R394W/R394W^* mice at E12.5 and E13.5. STRA8/DAZL double-staining experiment was performed with control (*Wt1^+/R394W^*) and *Wt1^R394W/R394W^* embryos at E11.5 (A–D), E12.5 (E–H), and E13.5 (I–L). Germ cells were labeled with DAZL (green). DAPI (blue) was used to stain the nuclei. The arrowheads point to STRA8-positive germ cells in *Wt1^R394W/R394W^* gonads. The gender of the embryos was confirmed with PCR using *Sry* primers.

**Figure 2:**
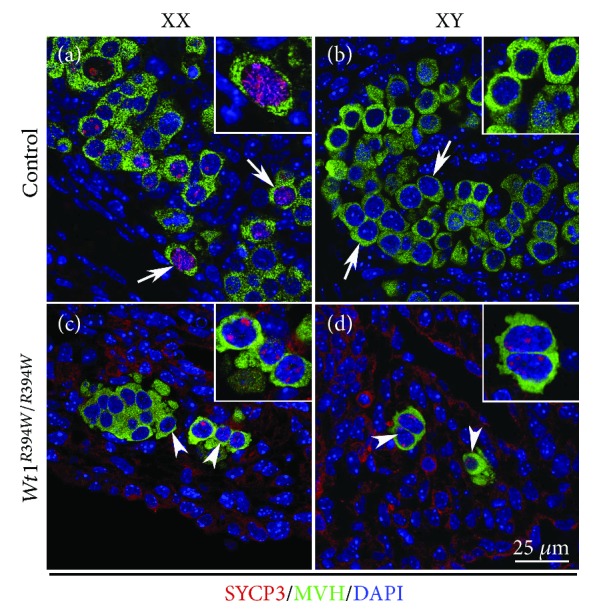
SYCP3 was expressed in germ cells of both male and female *Wt1^R394W/R394W^* gonads at E13.5. SYCP3/MVH double-staining experiment was performed with control *Wt1^+/R394W^* (A, B) and *Wt1^R394W/R394W^* (C, D) embryos at E13.5. Germ cells were labeled with MVH (green). DAPI (blue) was used to stain the nuclei. The arrowheads point to SYCP3-positive germ cells in *Wt1^R394W/R394W^* gonads. The gender of the embryos was confirmed with PCR using *Sry* primers.

Our previous study demonstrates that the genital ridge is absent in *Wt1^R394W/R394W^* mice due to the apoptosis of coelomic epithelial cells. However, the migration of PGCs is not affected and most germ cells arrive at position where genital ridge is formed [11]. To examine whether the differentiation of germ cells is affected in the absence of gonad somatic cells in *Wt1^R394W/R394W^* mice, the expression of meiotic genes STRA8 and SYCP3 was analyzed by immunofluorescence. As shown in Figure 1, STRA8 was expressed in germ cells of control ovaries at E12.5 (Figure 1A), and more positive germ cells were observed at E13.5 (Figure 1I). No STRA8 protein was detected in germ cells of control testes at E12.5 and E13.5 (Figure 1B, F, and J). In *Wt1^R394W/R394W^* mice, STRA8-positive germ cells were observed in both female (Figure 1G and K) and male gonads (Figure 1H and L) at E12.5 and E13.5. SYCP3 (synaptonemal complex protein 3), a lateral component of the synaptonemal complex, was first detected in control female germ cells at E13.5 (Figure 2A), but not in control male germ cells (Figure 2B). A few MVH/SYCP3 double-positive germ cells were noted in both male (Figure 2D) and female (Figure 2C) *Wt1^R394W/R394W^* gonads. In mammals, the timing of germ cell entry into meiosis is different between male and female. Female germ cell initiates meiosis right after sex determination. By contrast, male germ cell will not start meiosis during embryonic stage. Retinoic acid (RA) is a major extrinsic factor for germ cells to enter meiosis [15, 16]. *Stra8* is a gatekeeper gene for meiosis initiation which is expressed in germ cells in response to RA induction [3, 17]. In male gonad, meiosis is suppressed by RA-degrading enzyme CYP26b1 secreted from Sertoli cells during embryonic stage. In the present study, STRA8 and SYCP3 proteins were expressed in germ cells of both male and female *Wt1^R394W/R394W^* mice. These results indicated that RA is sufficient to induce STRA8 and SYCP3 expression in the absence of gonad somatic cells in both male and female gonads.

### 3.2. The Location of Germ Cells in Genital Ridge Was Disrupted when *Wt1* Was Inactivated at E9.5

**Figure 3:**
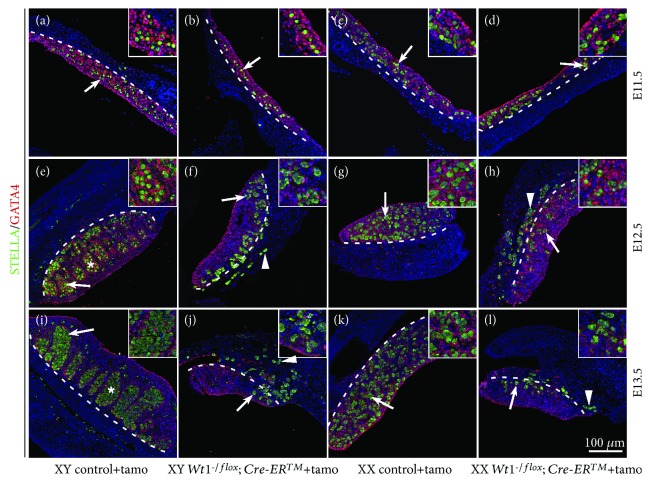
The location of germ cells was disrupted in *Wt1^-/flox^; Cre-ER^TM^* gonads. *Wt1^flox/flox^* females were crossed with *Wt1^-/flox^; Cre-ER^TM^* mice and the pregnant females were injected with tamoxifen at E9.5 to induce Cre activity. *Wt1^flox/flox^* and *Wt1^-/flox^* embryos were used as controls. Germ cells were labeled with STELLA (green, white arrows), and gonad somatic cells were labeled with GATA4 (red). The nuclei were stained in blue using DAPI. The dotted line denotes the border between the gonads and mesonephros. The arrowheads point to germ cells at the boundary between gonads and mesonephros. The gender of the embryos was confirmed with PCR using *Sry* primers. ^∗^Testicular cords.

**Figure 4:**
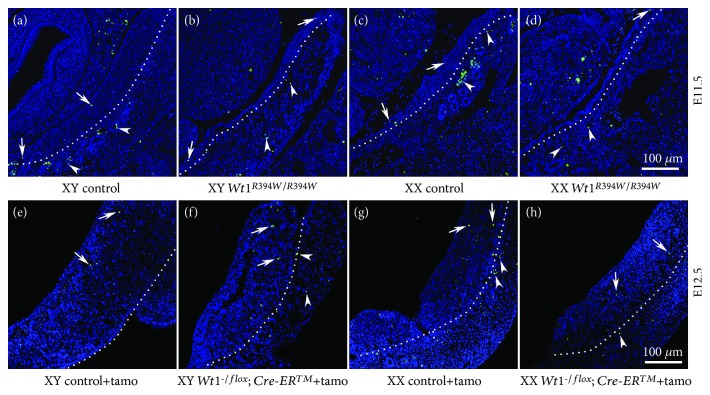
The number of apoptotic cells was not increased in *Wt1*-inactivated gonads. TUNEL assay was conducted in gonads of *Wt1* mutant (A, C: *Wt1^+/R394W^*; B, D: *Wt1^R394W/R394W^*) and *Wt1* knockout (E, G: *Wt1^flox/flox^* or *Wt1^-/flox^*; F, H: *Wt1^-/flox^; Cre-ER^TM^*) mice. Arrows and arrowheads point to apoptotic cells (green) in gonads and mesonephros, respectively. The nuclei were stained in blue using DAPI. The dotted line denotes the border between the gonads and mesonephros. The gender of the embryos was confirmed with PCR using *Sry* primers.

To further investigate the functions of somatic cells in germ cell development, *Wt1* was deleted at later stage using a tamoxifen-inducible Cre (*Cre-ER^TM^*) mice. *Wt1^flox/flox^* females were crossed with *Wt1^-/flox^; Cre-ER^TM^* males, and the pregnant females were injected with tamoxifen at E9.5 and the embryos were collected from E11.5 to E13.5. STELLA and GATA4 were used to label germ cells and somatic cells, respectively. As shown in Figure 3, the size of *Wt1*-deficient gonads (F, H, J, and L) was smaller than control gonads (E, G, I, K) at E12.5 and E13.5. In control males, the testicular cords were well organized at E12.5 and E13.5 (Figure 3E and I, asterisks). By contrast, no testicular cords were observed in *Wt1^-/flox^; Cre-ER^TM^* male gonads (Figure 3F and J), suggesting that *Wt1* is required for the testicular formation which is consistent with our previous study [18]. In control female gonads, germ cells were scattered inside the genital ridge (Figure 3G and K, white arrows). Interestingly, only a small portion of the germ cells were located inside the genital ridge of *Wt1^-/flox^; Cre-ER^TM^* mice at E12.5 and E13.5, and most germ cells were observed at the boundary between gonads and mesonephros (white dotted line). Our previous study has demonstrated that *Wt1* directs the lineage specification of Sertoli and granulosa cells. Without *Wt1* expression, the somatic cells differentiate into steroidogenic cells instead of supporting cells [12]. In this mouse model, *Wt1* was deleted at E10.5 approximately. The abnormal differentiation of supporting cells became evident after E11.5^12^ and the mislocation of germ cells in *Wt1^-/flox^; Cre-ER™* mice was observed at E12.5 and E13.5. Based on these results, we speculated that structure support or paracrine signals released from somatic cells are indispensable for the precise location of germ cells in the gonads. However, the detailed regulatory mechanism needs further investigation. To further examine whether abnormal differentiation of gonad somatic cell causes germ cell death, TUNEL assay was performed. As shown in Figure 4, a small number of TUNEL-positive cells (green) were observed in both control and *Wt1*-inactivated gonads, and no significant difference was noted between control and *Wt1^R394W/R394W^* gonads (A–D) or control and *Wt1^-/flox^; Cre-ER^TM^* gonads (E–H). These results indicated that aberrant differentiation of gonad somatic cells does not cause germ cell death.

### 3.3. The Expression of Meiosis-Associated Genes in Both Male and Female Germ Cells of *Wt1*-Inactivated Gonads

**Figure 5:**
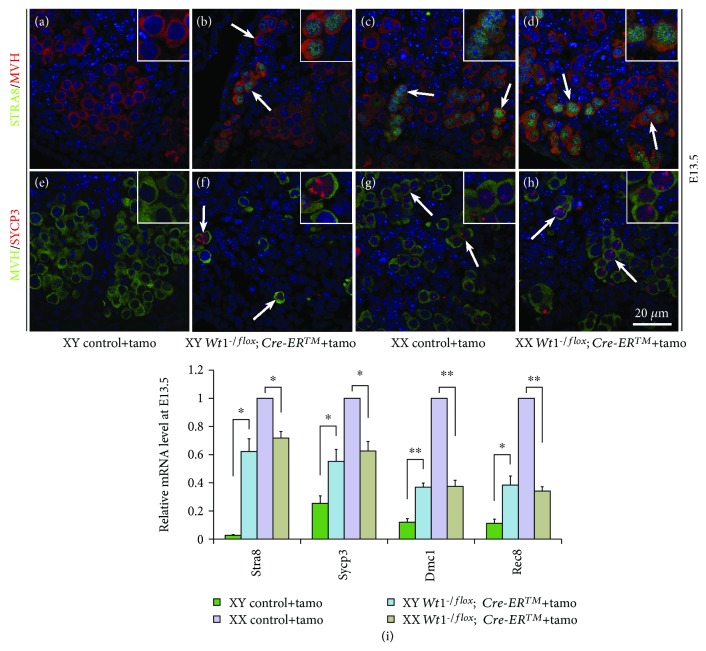
The meiotic genes were expressed in germ cells of both male and female *Wt1^-/flox^; Cre-ER^TM^* mice after tamoxifen induction. *Wt1^flox/flox^* females were crossed with *Wt1^-/flox^; Cre-ER^TM^* mice, and the pregnant females were injected with tamoxifen at E9.5 to induce Cre activity. *Wt1^flox/flox^* and *Wt1^-/flox^* embryos were used as controls. A–H: immunofluorescence analysis of STRA8/MVH (A–D) and SYCP3/MVH (E–H) in control and *Wt1^-/flox^; Cre-ER^TM^* embryos at E13.5. Germ cells were labeled with MVH. DAPI (blue) was used to stain the nuclei. The arrows point to double-positive germ cells. I: real-time PCR analysis of *Stra8*, *Sycp3*, *Dmc1*, and *Rec8* in control and *Wt1^-/flox^; Cre-ER^TM^* embryos at E13.5. *Hprt1* was used as an endogenous control. The data are presented as mean ± SEM. ^∗^*P* < 0.05; ^∗∗^*P* < 0.01.

To test whether the differentiation of germ cells is affected in *Wt1^-/flox^; Cre-ER^TM^* gonads, the expression of STRA8 and SYCP3 was examined by immunofluorescence. As shown in Figure 5, STRA8 (A) and SYCP3 (E) proteins were not expressed in germ cells of control males at E13.5. However, both STRA8 (Figure 5B and D) and SYCP3 (Figure 5F and H) signal were detected in germ cells of *Wt1^-/flox^; Cre-ER^TM^* male and female gonads at E13.5. The expression pattern resembled that of the control female germ cells (Figure 5C and G). The mRNA level of meiosis-associated genes was also analyzed by real-time PCR. The expression of *Stra8*, *Sycp3*, *Dmc1* (a meiosis specific recombinase), and *Rec8* (a meiotic cohesin) was similar between male and female *Wt1^-/flox^; Cre-ER^TM^* gonads at E13.5, and it was significantly increased compared to the control male gonads (Figure 5I).

**Figure 6:**
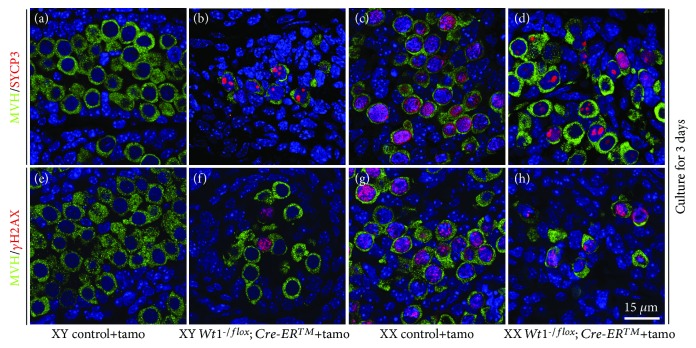
The meiosis was blocked in germ cells of *Wt1^-/flox^; Cre-ER^TM^* gonads. *Wt1^flox/flox^* females were crossed with *Wt1^-/flox^; Cre-ER^TM^* mice, and the pregnant females were injected with tamoxifen at E9.5 to induce Cre activity. Control (*Wt1^flox/flox^* and *Wt1^-/flox^*) and *Wt1^-/flox^; Cre-ER^TM^* gonads were dissected at E13.5 and cultured in vitro for 3 days. MVH/SYCP3 (A–D) and MVH/*γ*H2AX (E–H) double-staining experiment was performed, and germ cells were labeled with MVH (green). DAPI (blue) was used to stain the nuclei. The gender of the embryos was confirmed with PCR using *Sry* primers.

Because most *Wt1^-/flox^; Cre-ER^TM^* embryos died at E14.5 after tamoxifen induction, to examine the meiosis of germ cells at later developmental stage, control and *Wt1^-/flox^; Cre-ER^TM^* gonads with mesonephroi were dissected at E13.5 and cultured in vitro for 3 days. The expression of SYCP3 and *γ*H2AX (phospho-H2AX histone) was examined by immunofluorescence. As shown in Figure 6, thread-like SYCP3 signal was observed in germ cells of the control ovaries and no SYCP3 signal was detected in germ cell of the control testes. The expression of SYCP3 was also detected in germ cells of both *Wt1^-/flox^; Cre-ER^TM^* male and female gonads. However, only scattered SYCP3 foci were noted. The expression of *γ*H2AX protein which marks DNA double-strand breaks was also detected in germ cells of both *Wt1*-deficient male and female gonads, but the number of *γ*H2AX-positive germ cells was significantly reduced compared to the control females.

**Figure 7:**
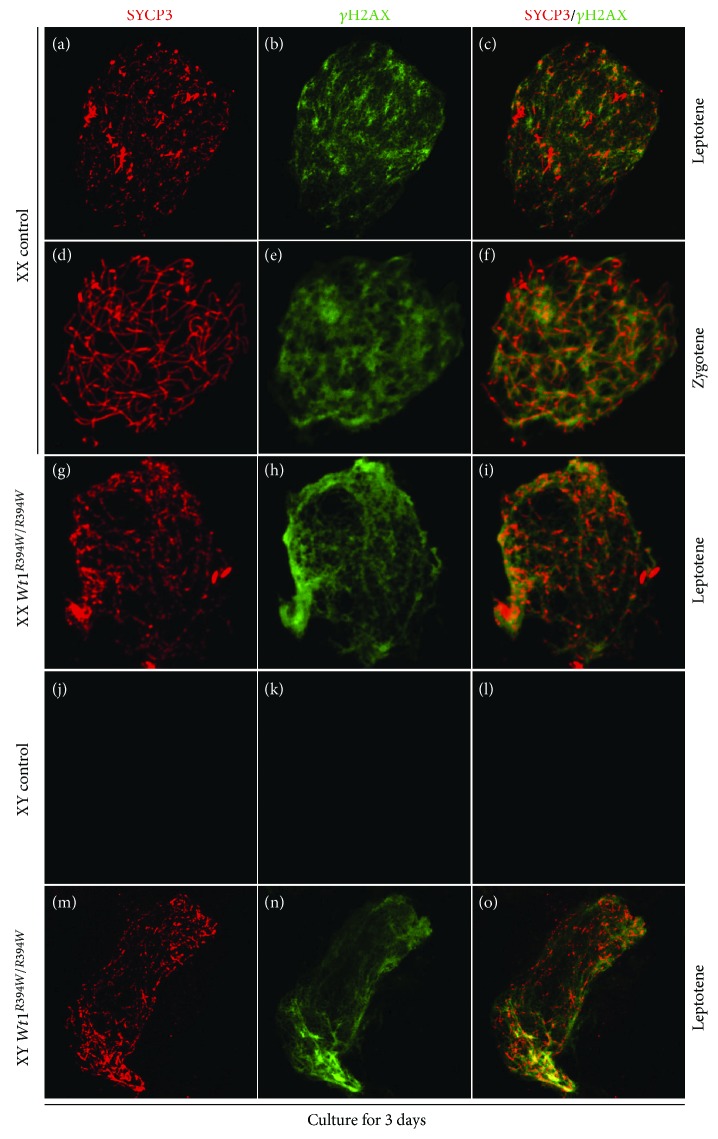
Meiosis was blocked at leptotene stage in both male and female germ cells of *Wt1^R394W/R394W^* gonads. Immunofluorescent staining of SYCP3 (red) and *γ*H2AX (green) were performed on chromosome spreads of control (*Wt1^+/R394W^*; A–F: female; J–L: male) and *Wt1^R394W/R394W^* (G–I: female; M–O: male) gonads which were dissected at E12.5 and cultured in vitro for 3 days. The gender of the embryos was confirmed with PCR using *Sry* primers.

To further confirm the results, the gonads with mesonephroi of control and *Wt1^R394W/R394W^* mice were also dissected at E12.5, cultured in vitro for 3 days, and then subjected to chromosome spreads and immunofluorescence of SYCP3 and *γ*H2AX. In the control female ovaries, most germ cells have progressed to zygotene stage (Figure 7D–F) and a few germ cells were in leptotene (Figure 7A–C). However, in both male (Figure 7M–O) and female (Figure 7G–I) *Wt1^R394W/R394W^* germ cells, meiosis cannot progress beyond leptotene stage.

SYCP3 is a lateral element of synaptonemal complex that forms between two homologous chromosomes. Its localization pattern during meiosis is used to identify cells at different stages of meiotic prophase I. SYCP3 first appears diffusely in the leptotene stage. As meiosis progresses to the zygotene stage, SYCP3 forms line-shaped structure. In late zygotene and pachytene, prominent synapsis marked by SYCP3 is observed [19]. Although SYCP3 protein were expressed in the germ cells of *Wt1*-inactivated mice, only diffuse signal was observed, suggesting the meiosis is not properly initiated.

In this study, we found that STRA8 and SYCP3 were expressed in the male germ cells of both *Wt1^R394W/R394W^* and *Wt1^-/flox^; Cre-ER^TM^* mouse models. A possible reason for this phenomenon is that the Sertoli cell differentiation was blocked and no testicular cords were formed in these mouse models. Therefore, the expression of CYP26b1 was decreased and the germ cells would access mesonephros-derived RA, which in turn induced the expression of STRA8 and SYCP3. It has been demonstrated that primordial germ cells at E11.5 are bipotential and they enter meiosis or not depending on the somatic cells [20]. Interestingly, only scattered SYCP3 signals were observed in both male and female germ cells of *Wt1*-deficient gonads. These results indicate that RA is not sufficient to induce germ cell meiosis initiation and somatic cell environment is also essential for normal meiosis process.

## 4. Conclusions

Our study demonstrates that aberrant differentiation of somatic cells leads to abnormal meiosis in both male and female germ cells. RA is not sufficient to induce germ cell meiosis initiation, and somatic cell environment is also essential for normal meiosis process.

## Data Availability

The data used to support the findings of this study are included within the article.
